# Phase-sensitive detection of anomalous diffusion dynamics in the neuronal membrane induced by ion channel gating

**DOI:** 10.1088/1361-6560/acbf9c

**Published:** 2023-03-13

**Authors:** Honggu Choi, Rishyashring R Iyer, Carlos A Renteria, Stephen A Boppart

**Affiliations:** 1 Beckman Institute for Advanced Science and Technology, University of Illinois at Urbana-Champaign, Urbana, United States of America; 2 Department of Electrical and Computer Engineering, University of Illinois at Urbana-Champaign, Urbana, United States of America; 3 Department of Bioengineering, University of Illinois at Urbana-Champaign, Urbana, United States of America; 4 Carle Illinois College of Medicine, University of Illinois at Urbana-Champaign, Urbana, United States of America; 5 Cancer Center at Illinois, University of Illinois at Urbana-Champaign, Urbana, United States of America

**Keywords:** anomalous diffusion, neuron imaging, neuron dynamics, digital holography, memory effect, ion gating

## Abstract

Non-ergodicity of neuronal dynamics from rapid ion channel gating through the membrane induces membrane displacement statistics that deviate from Brownian motion. The membrane dynamics from ion channel gating were imaged by phase-sensitive optical coherence microscopy. The distribution of optical displacements of the neuronal membrane showed a Lévy-like distribution and the memory effect of the membrane dynamics by the ionic gating was estimated. The alternation of the correlation time was observed when neurons were exposed to channel-blocking molecules. Non-invasive optophysiology by detecting the anomalous diffusion characteristics of dynamic images is demonstrated.

## 1. Introduction

Neurons have rapidly alternating cellular dynamics that are induced by intermittent gating of ion channels embedded in the cellular membrane, and these cellular dynamics are far from being characterized by thermal diffusion (Sikora *et al* 2017). The origin of the electric activity of neurons is from transmembrane ion transport and gating, which has been investigated extensively by electrophysiology (Sakmann and Neher 1984, Chow and White 1996, White *et al* 2000). While this method can demonstrate the precise dynamic characteristics of ion transport and gating, the measurement only characterizes the localized channels with an invasive tip from a micropipette. Deploying multiple micropipette electrodes could enable one to observe the activities of multiple neurons (Shein *et al* 2009), however, the micropipette electrodes can generate artifacts (Anikeeva *et al* 2012), the number of electrodes per unit area can be limited, and culturing and controlling the growth of neurons at the desired location and interconnectivity remain an issue. Optical detection and imaging of neuronal dynamics is one class of approaches to realize a non-invasive and non-local measurement from neurons in culture.

In previous studies, optical detection of neuronal activity was performed by intensity or phase fluctuation of neurons (Lazebnik *et al* 2003, Graf *et al* 2009, Akkin *et al* 2010, Ling *et al* 2018, Ling *et al* 2020, Renteria *et al* 2020, Iyer *et al* 2022). In particular, a phase-sensitive modality could directly observe the geometric or refractive index changes within the neurons by the deformation of the wavefront that can be analyzed by random walk models. The use of optical coherence microscopy (OCM) for phase-sensitive detection provides quantitative information on the optical thickness and/or refractive index changes of neurons, and ultrafast sampling enables the detection of neuronal activities from and between multiple neurons simultaneously. Faster sampling, however, results in a lower signal-to-noise ratio by the reduced photon flux, thereby limiting the accurate estimation of phase (Hosseini *et al* 2016). A common way to estimate the dynamic contrast of dynamic phase measurements and any associated noise is by acquiring the variance of the phase (Mahmud *et al* 2013). The variance represents a diffusion coefficient by assuming a Markovian process with no memory, which is not a rigorous approach for non-equilibrium and non-ergodic systems. The non-ergodicity, the disagreement of the ensemble, and the temporal averages make the physical interpretations of the statistics challenging, as these violate the fluctuation-dissipation theorem (Lapas *et al* 2007). To interpret the random dynamics of a non-ergodic system, a memory effect has been introduced which is defined as a correlation time of the driving force by the second fluctuation-dissipation theorem that represents how long a random motion lasts (Kubo 1966).

Anomalous diffusion of biological systems with the memory effect has been widely observed in microscopic to macroscopic scales such as protein molecular dynamics, intracellular dynamics, ionic gating dynamics, immune responses, and cortical networks (Mercik *et al* 1999, Mercik and Weron 2001, Min *et al* 2005, Cook *et al* 2014, Lisowski *et al* 2015, Han *et al* 2020, Choi *et al* 2021b, Dieterich *et al* 2022). The advantage of studying anomalous diffusion is that such dynamics in various systems with dramatically different scales are often scale invariant that can be expressed as scale-free power-law correlations by the memory effect (Marinari *et al* 1998, Cavagna *et al* 2010, Munoz-Gil *et al* 2021). The memory effect of the colored noise without a DC component of the system can induce anomalous diffusion (Morgado *et al* 2002). The ionic exchanges that occur in the cellular membrane are known to be colored noise, which can be the source of the memory effect and induce membrane fluctuations with anomalous diffusion characteristics (Mercik *et al* 1999, Mercik and Weron 2001, Schmid *et al* 2006). Colored noise plays an important role known as stochastic resonance in non-Markovian systems (Goychuk and Hanggi 2003a), for instance, a network formed by multiple neurons having non-ergodic interactions could have enhanced communications by the noise (Ushakov *et al* 2010, Surazhevsky *et al* 2021). The investigation of the memory effect of such noise driving neuronal dynamics requires a simultaneous spatial and temporal measurement method without contacting or labeling neurons to minimize artifacts (Drapaca 2021), which remains challenging. Ultrafast phase-sensitive optical imaging systems have demonstrated the correlation between the electric activities of cells and the corresponding membrane fluctuations (Ling *et al* 2020, Iyer *et al* 2022), where the relationship between the electric activity and the non-Markovian characteristics can be further investigated. The driving force of the system can be classified as internal and external noises. When the fluctuation and dissipation are from the same source, then the system is driven by internal noise and when they are not from the same source, then the driving force is external noise (Wang and Tokuyama 1999).

In the case of neuronal communications, the interaction with neighboring neurons can occur spontaneously or by stimulations externally (Chow and White 1996, Paul *et al* 2017), and the estimation of such internal and external noise contributions would provide a better understanding of the neuronal communications. Here, the neuronal membrane dynamics were observed by phase-sensitive OCM, and the characteristics of anomalous diffusion in the membrane were analyzed. The driving force that causes conformation changes in a neuronal membrane was assumed to originate from ionic exchanges (Schmid *et al* 2006). The ionic gating dynamics have been reported to have a memory effect (Mercik *et al* 1999, Mercik and Weron 2001, Pfeiffer *et al* 2020), and the driving force is also expected to have a memory effect as the force is proportional to the ion flux. Note that the DC component of the noise power spectrum that drives the system determines whether the process is anomalous diffusion or not (Morgado *et al* 2002), and the DC component was assumed to be zero in our experiment as the net sizes and positions of cells did not change during the time frame of measurements (∼2.5 s).

From the anomalous diffusion characteristics, the memory effect of the noise induced by the ionic exchange was estimated. The fluctuations of neuron membrane had Lévy-like distributions which can be correlated to systems with non-ergodic and non-equilibrium dynamics whose random walks showed Lévy walks, and the corresponding memory effect can be further estimated (Lutz 2004, Margolin and Barkai 2006, Rebenshtok and Barkai 2008). The suppression of the driving force on the membrane by blocking ion channels changed the anomalous diffusion characteristics of the fluctuations. The distribution of the fluctuations showed Lévy-like distribution with a power-law tail while the ionic exchange enabled, and it became a Gaussian-like (no power-law tail) distribution after the suppression which represented the ion exchanges contributed to the non-Markovian characteristics of the membrane fluctuations (Baeumer and Meerschaert 2010, Cherstvy *et al* 2013, Liemert *et al* 2017, dos Santos 2019). The temporal asymptotic form of the memory effect of neurons was further estimated from the fractional exponent of the Lévy-distribution (Deering and West 1992, Kantelhardt *et al* 2002) and the corresponding correlation time of the electric activity was estimated.

## 2. Materials and methods

### 2.1. Neuron preparation and the measurements of the neuronal dynamics

**Figure 1.**
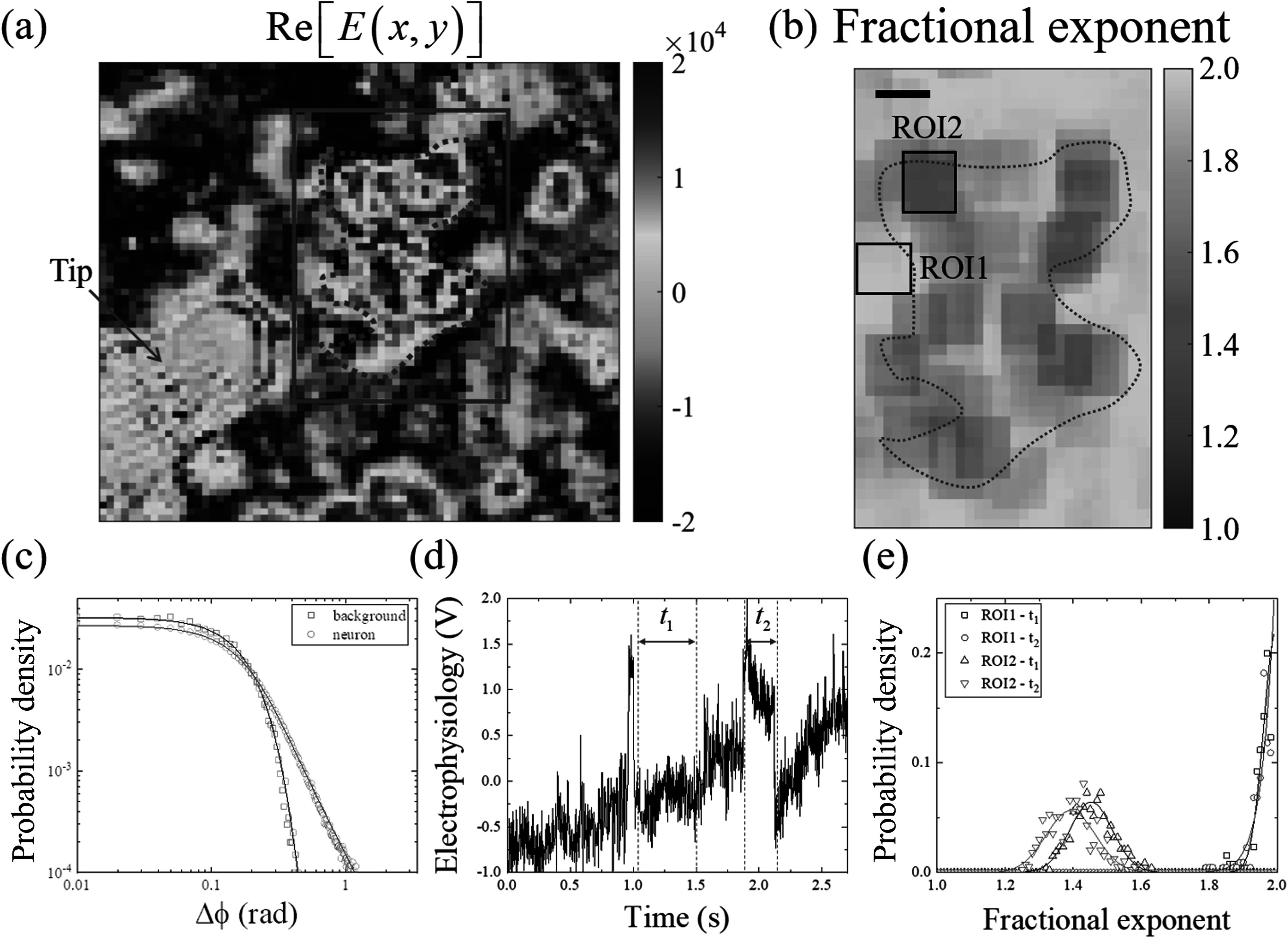
Phase-sensitive optical coherence microscopy images and the dynamics related to electrical activity in neurons. (a) The real component of the reconstructed digital holographic image ($\mathrm{Re}\left[E(x,y)\right]$) of the field of view (arb. units). The image of a cluster of neurons (the cellular boundary marked by a red dashed curve within red rectangular), and (b) the corresponding fractional exponent *(α*) map of 25 ms. Two regions of interest include the background (ROI1) and the neuron (ROI2), which were selected for the analyses. Scale bars in (a) and (b) represent 3 *μ*m. (c) The estimation of *α* values from the probability density functions (PDF) of optical displacements (Δϕ) of ROI1 and ROI2 in 25 ms. The power-law features of the neuron and background are compared to Lévy distributions (Solid lines). The PDF acquired from the background (ROI1, *α* = 1.99) and the neuron (ROI2, *α* = 1.44) show distinct power-law distributions. (d) The electrophysiology signal acquired by the micropipette electrode tip in (a). The inactive time (*t*_*1*_) and the active time (*t*_*2*_) segments were selected and the corresponding *α* values were collected from ROI1 and ROI2, respectively. (e) The distributions of *α* values during the time segments *t*_*1*_ and *t*_*2*_. The systematic increase or decrease of *α* values can be related to the electrophysiology signal generation from the neurons.

The neuronal stem cells, the NE-4C cell-line that was driven from the anterior brain vesicles of E9 mouse embryos, were selected as a neuron model as this line was close to the early stage of neuroectodermal progenitors and known to have a −70 mV membrane potential (Schlett and Madarasz 1997, Jelitai *et al* 2007). The differentiated NE-4C neuron cells (CRL-2925, American Type Culture Collection, Manassas, VA, USA ∣ RRID:CVCL_B063) were cultured in Eagle’s modified essential growth medium with 4 *μ*M of L-glutamine (10009CV, Corning, Corning, NY, USA), 10% fetal bovine serum (16140071, Thermo Fisher Scientific, Waltham, MA, USA), and 1% penicillin-streptomycin (10378016, Thermo Fisher Scientific) for 30 h. After 30 h, 1 *μ*M all-trans retinoic acid carried by 0.01% DMSO was added. The medium was refreshed every day. The grown cells were transferred to a poly-d-lysine coated plate on day 7. When a cultured dish was prepared, the dish was mounted on a stage and the spontaneous neuronal activities were recorded by OCM. The configuration of the OCM imaging system is an off-axis Mach–Zehnder-type interferometer that collects light reflected from cultured neurons to measure the optical thickness and/or change in the refractive index of neurons (Iyer *et al* 2022). The optical configurations and the set-up to acquire the phase-sensitive images were identical to the previous report from Iyer *et al* (2022), but only used a horizontal polarization component. The illumination source is a superluminescent diode at *λ* = 865 ± 65 nm (Broadlighter S860-HP, Superlum Inc., Cork, Ireland) and an ultrafast camera (4000 Hz, Mini AX100, Photron, Tokyo Japan) was used. The coherence gate was located at the dish surface to measure the optical thickness of neurons as the neuron cells were transparent. The optical thickness of the neuron cluster (both cell bodies and axons) with a proximate electrophysiology micropipette electrode tip is shown in figure 1(a) acquired from the phase angle of coherently reconstructed complex-valued images. The coherent reconstruction of the complex-valued image was performed by the angular de-modulation of a hologram. A single pixel represents 0.5 *μ*m. The fluctuation of the optical thickness was measured by acquiring the displacement of the phase angle between consecutive frames. The images were recorded for 2.5 s (10^4^ frames) and the spontaneous electrical activity of the neuron cluster was measured by the micropipette electrode, simultaneously. The optical displacements between frames were estimated by dividing consecutive complex-valued frames and acquiring the displacement of phase angle (*Δϕ*) closest to zero to reject phase-wrapping artifacts (Choi *et al* 2021a). The value *Δϕ* represents the optical displacement for 0.25 ms and the probability density function (PDF) of *Δϕ* was acquired for 25 ms (10^2^ frames).

### 2.2. Estimation of the memory effect of the neuronal membrane fluctuations by electric activity

The general estimation of the anomalous diffusion is made by acquiring the mean squared displacement (MSD). The ensemble average of MSD has the asymptotic form $\langle {x}^{2}\left(t\right)\rangle \sim {t}^{2H}$ for a free particle, where *H* is a Hurst exponent with $0< H< 1/2$ and $1/2< H< 1,$ which represents the range of Hurst exponents for subdiffusion and superdiffusion processes, respectively (Mandelbrot and Vanness 1968). The estimation of the MSD for the cellular membrane is not an appropriate approach as the system is bounded and whose asymptotic form of MSD is not ∼*t* ^2*H*^ (Vinales and Desposito 2006, Di Terlizzi *et al* 2020). Also, ultrafast biological processes such as neuronal activity occur within the order of a millisecond, therefore, the number of data points is insufficient to estimate the asymptotic power-law feature of the MSD even with an ultrafast camera. However, using the relationship between the Hurst exponent and the fractional exponent of the Lévy distribution, known as $H=1/\alpha ,$ enables estimating the fractional exponent of the memory effect (Kantelhardt *et al* 2002). The asymptotic form of the memory effect *γ(t)* originated from the force induced by ionic exchanges can be estimated from the Hurst exponent as a correlation time *C(t)* of the internal noise: $C\left(t\right)={k}_{{\mathrm{B}}}T\gamma \left(t\right)\sim {t}^{-2H}$ at temperature *T* with the Boltzmann constant *k*_B_, by the second fluctuation-dissipation theorem (Wang and Tokuyama 1999, Vinales and Desposito 2006, Maes 2014). From the shape of experimentally acquired PDFs that had Lévy distributions, we estimated the corresponding fractional exponent by comparing the power-law tails. The memory effect of the membrane fluctuation was estimated where the values of the Hurst exponent (*H*) or the fractional exponent (*α*) was 0.5 or 2, respectively; then the fluctuation had no memory whose PDF was Gaussian-like, while for *H* = 1 or *α* = 1, the PDF was Cauchy-like and we estimated the fluctuation was ballistic.

The physical displacement *Δz* of the neuronal membrane estimated from the optical displacement is ${\mathrm{\Delta }}z={\mathrm{\Delta }}\phi \lambda /4\pi n,$ where *n* is the refractive index of the medium (*n* ∼ 1.33) and *λ* is the center wavelength of OCM. The membrane displacements were assumed to obey the Lévy process that states that equal displacements have equal probability and the displacements of each temporal step are independent. A single neuron was expected to have the same Lévy process and the same Lévy distribution of the membrane displacement. The PDF acquired from the optical displacements of a cellular membrane has a stable and symmetric Lévy-like distribution with the fractional exponent *α*, and the scaling factor *γ* (Mantegna 1994):

\begin{eqnarray*}{L}_{\alpha ,\gamma }\left({\mathrm{\Delta }}\phi \right)=\displaystyle \frac{1}{\pi }\displaystyle {\int }_{0}^{\infty }{e}^{-\gamma {q}^{\alpha }}\,\cos \left(q{\mathrm{\Delta }}\phi \right)dq.\end{eqnarray*}

The distribution becomes Cauchy (ballistic process) or Gaussian (diffusion process) when $\alpha =1,2,$ respectively, which is consistent with the Hurst exponent estimation of $H=1$ for a Cauchy ballistic process and $H=0.5$ for a Gaussian diffusion process. The fractional exponent of the PDF ($p\left({\mathrm{\Delta }}\phi \right)$) was estimated by the maximum-likelihood estimator *l*_*α*,*γ*_ (MLE) of Lévy distribution *L*_*α*,*γ*_ in equation (1) (Clauset *et al* 2009, Choi *et al* 2022)

\begin{eqnarray*}{l}_{\alpha ,\gamma }=\displaystyle \sum _{{\mathrm{\Delta }}\phi =-\pi }^{\pi }p\left({\mathrm{\Delta }}\phi \right)\mathrm{log}\,{L}_{\alpha ,\gamma }\left({\mathrm{\Delta }}\phi \right).\end{eqnarray*}

The *α* and *γ* values of a PDF were determined when the MLE (*l*_*α*,*γ*_) value was at maximum. The goodness of the estimation was compared to the shape of power-law tails and the value of the fractional exponent and the estimated uncertainty of the fractional exponent value was ±0.01. The neuron cluster, background, and the corresponding *α* value map are shown in figure 1(b), and as an example, the PDFs acquired from the two regions of interest (background and neurons) and the MLE of the Lévy distributions are compared in figure 1(c). For accurate estimations of the *α* value of a PDF, the membrane displacements were collected from an area close to a single cellular dimension, assuming the dynamics of a membrane had the same Lévy process. As the spontaneous neuronal dynamics varied rapidly over time, the size of the time window was kept as small as possible, at 25 ms (10^2^ images), by sacrificing the image resolution (from 0.5 to 3 *μ*m) to maintain the sample size at 3.6 × 10^3^. The ensemble of displacements was collected from a tile close in dimension to that of an axon of a neuron (3 *μ*m by 3 *μ*m, 6 pixels by 6 pixels). A total of 36 pixels (a 6-pixel-by-6-pixel square tile) in each of 100 frames were used to acquire a PDF and estimate the fractional exponent of a single pixel index. The fractional exponent map shown in figure 1(b) was acquired from the fractional exponents of the corresponding PDFs, taken from oversampled ensembles by shifting the tile location by 0.5 *μ*m horizontally or vertically.

## 3. Results and discussion

To investigate the correlation between the spontaneous neuronal activity in figure 1(d) and the power-law tails of the PDFs, the collection of *α* values from the regions of interest 1 and 2 (ROI1 and ROI2, black rectangular in figure 1(b)) during electrically inactive (*t*_*1*_) and active (*t*_*2*_) time segments are compared in figure 1(e). The systematic shift of the distributions of *α* values was observed, which represents the fluctuation of the *α* values, and the decrease of *α* over time represents spontaneous neuronal activity.

Neurons at their resting potential still undergo spontaneous low-level ion exchanges due to current leakage and active transport for their homeostasis (Spruston and Johnston 1992, Chow and White 1996, Vergara *et al* 2019), and subsequently, no dramatic shift of *α*-value distribution was observed in figure 1(e). ROI2 showed a Gaussian distributions (*α*–2) as the measurement was perturbed by white noises from the optical intensity fluctuations and mechanical vibrations (Choi *et al* 2021a, Choi *et al* 2022).

**Figure 2.**
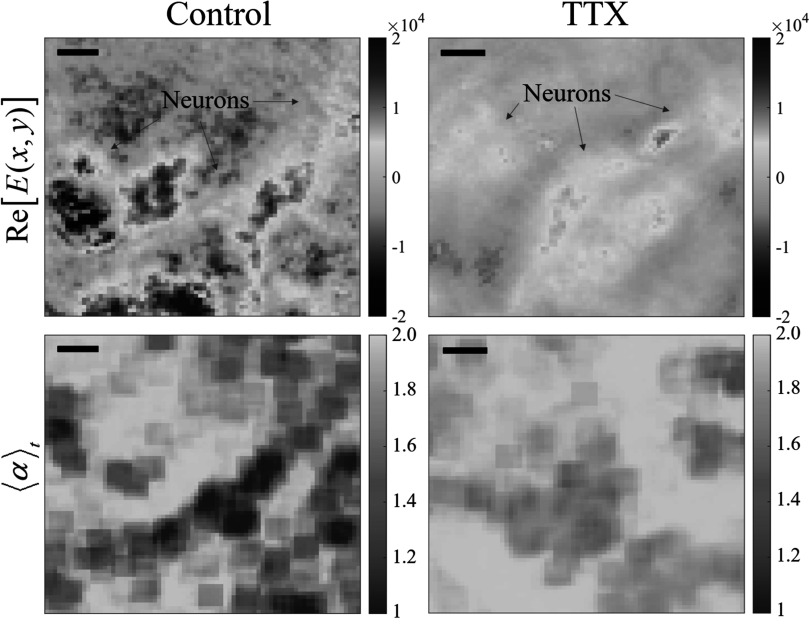
Reconstructed holographic images of neuron cultures, including both control cultures and those exposed to tetrodotoxin (TTX, 100 nM) (top row), along with the corresponding time-averaged (2.5 s) fractional exponent (${\langle \alpha \rangle }_{t}$) maps (bottom row). The ${\langle \alpha \rangle }_{t}$ values of the neuronal cluster and the background show contrast for the control culture, while the culture of neurons exposed to TTX shows weak contrast. Scale bars represent 5 *μ*m.

For further validation of the relationship between the *α* values and ion gating, tetrodotoxin (TTX, 100 nM) was applied to the cultured neurons and incubated for 20 min When neurons are exposed to TTX molecules, sodium channels are blocked, while preserving other cellular dynamics, such as those related to glucose metabolism (Shibata and Moore 1993, Bane *et al* 2014). The reconstructed images of representative cases are shown in figure 2. The dynamics of neurons were recorded and analyzed (Supplemental video file).

**Figure 3.**
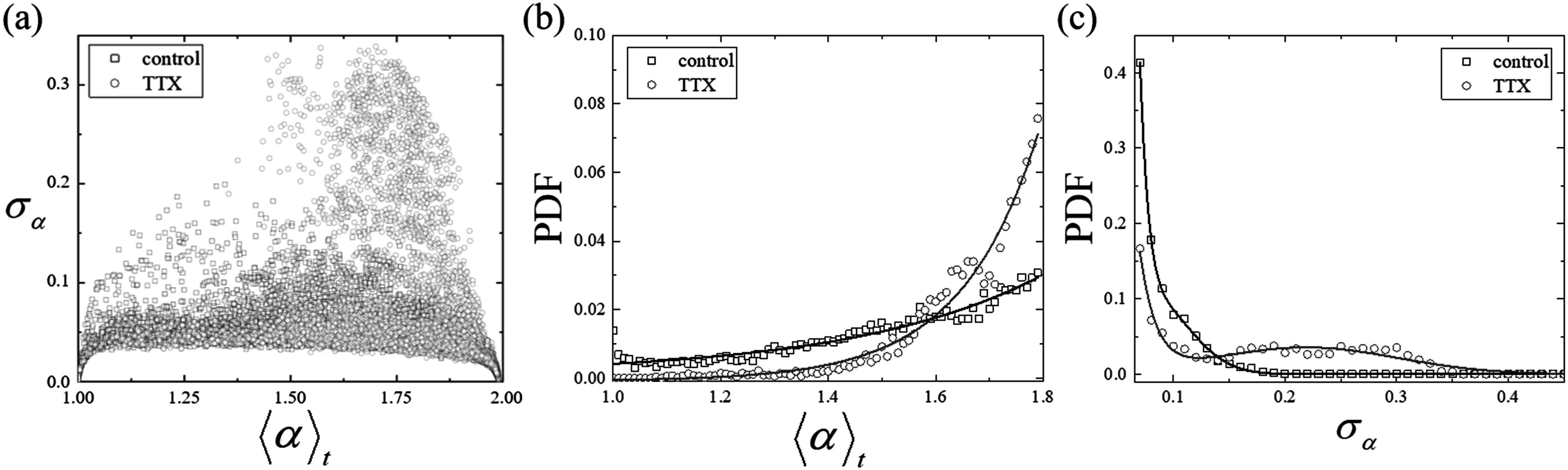
Quantitative comparison of the fractional exponent distribution between control and TTX-exposed neuron cultures. (a) Scatter plot of ${\langle \alpha \rangle }_{t}$ and the corresponding standard deviation (${\sigma }_{\alpha }$) of fractional exponents collected from 6 independent neuron cultures. (b) PDF of ${\langle \alpha \rangle }_{t}$ acquired from control (sample size: *N* = 3) and TTX-exposed (*N* = 3) cultures. Two exponentially decaying distributions with different decaying coefficients were observed. The probabilities of observing ${\langle \alpha \rangle }_{t}$ below 1.4 were 26% and 3% for the control and TTX-exposed cultures, respectively. The probabilities were normalized by the sum of distributions between $1\leqslant \alpha \leqslant 1.8.$ The guidelines are exponentially decaying curves. (c) The distribution of ${\sigma }_{\alpha }.$ The hump between 0.1 and 0.4 is from the distinct distribution in (a). The guidelines represent two-Gaussian estimations.

From the fractional exponent maps shown in figure 2, the memory effect of the driving force by the ion channel gating can be estimated. The systematic increases in the fractional exponent that were induced by TTX were also observed. For a quantitative comparison, the fractional exponent distributions of 6 independent neuron cultures were acquired, from both control cultures (sample size: *N* = 3) and TTX-exposed cultures (*N* = 3). The time-averaged fractional exponent values over 2.5 s (${\langle \alpha \rangle }_{t}$) and the corresponding standard deviation of *α* values (*σ*_*α*_) were acquired from the entire field-of-view of the 6 cultures and are shown in figure 3(a). The *σ*_*α*_ value can be related to the consistency of the memory effect. The memory effect was preserved in the control group, on the other hand, the memory effect was suppressed and/or inconsistent (*α* and/or *σ*_*α*_ values became larger) when the neurons were exposed to TTX. The distribution in figure 3(a) was normalized between $1\leqslant \alpha \leqslant 1.8$ in figure 3(b) as the fractional exponent values shown in figure 1(e) suggest that the values higher than 1.8 are close to the signal from the background. The *σ*_*α*_ values of the control and TTX-exposed cultures are compared in figure 3(c), which were normalized with the values $0.07\leqslant {\sigma }_{\alpha }$ to emphasize the difference. The probability of neurons having lower than *α* = 1.4 was 26% for the control group, while that for the TTX-exposed group was only 3%. The increase in ${\langle \alpha \rangle }_{t}$ represents the decrease in the the memory effect which can be ultimately related to the decrease in the correlation time of ion gating, which is consistent with the mechanism of TTX. The hump of *σ*_*α*_ between 0.1 and 0.4 in figure 3(c) is likely the result of intermittent ion exchanges other than Na^+^ channels in the presence of TTX, which are known to have slow responses (Kostyuk *et al* 1981). For instance, Ca^2+^ channels have a slow repolarization time (∼100 ms) (Randall and Tsien 1997). However, TTX exposure inhibits the generation of action potentials, as the sodium channels are the primary driving channels for these.

Based on these demonstrated results, we further estimated the memory effect from the fractional exponent. The memory effect is described in an asymptotic form of $\gamma (t)\sim {t}^{-2H},$ which is a temporal correlation of the driving force. Acquiring the fractional exponent could be the related to the correlation time of the force induced by the ionic gating (Kubo 1966, Kantelhardt *et al* 2002). The conformational diffusion model of ion channel gating predicts a closing time that has a power-law feature and the corresponding neuronal conductance has been estimated (Goychuk and Hanggi 2003b, 2004). The induced driving force by ion gating is proportional to the temporal population of open channels. The induced ionic current can be estimated by the correlation times of the closing time and opening time. The memory effect of the ionic current was estimated by the power-law dwelling times that showed a fractional-exponent memory kernel (Mercik *et al* 1999). Assuming that the stochastic process of the neuronal dynamics is self-similar (Mandelbrot and Vanness 1968), and the ionic current by gating is *I(t)*, then the autocorrelation function *κ(t)* of *I(t)* by ion gating can be represented with the fractional exponent value as (Mercik *et al* 1999)

\begin{eqnarray*}\kappa \left(\tau \right)=\displaystyle \frac{\langle I\left(t\right)I\left(t+\tau \right)\rangle -{\mu }^{2}}{{\sigma }^{2}}\propto {\tau }^{-2\left(1-1/\alpha \right)}.\end{eqnarray*}

Here, *μ* and *σ* are the mean and standard deviation of the ionic current, respectively. From the autocorrelation function, we can further estimate the PDF of the closed-time distribution whose asymptotic power-law feature is ${t}^{-\left(3-2/\alpha \right)}$ (Mercik and Weron 2001). Also, the Hurst exponent can predict the fractional geometry (*d*) of the dynamics as $d=2-H$ for the topological 1D space, and the long-term dependence of the power spectrum $s\left(\omega \right)\sim {\omega }^{-1-2H}$ (Reed *et al* 1995). The fractional geometry represents the smoothness of a dynamic signal that might be further understood as the detection feasibility of membrane dynamics by neuronal activity (Deering and West 1992).

The conserved memory effect for the control group and the reduced memory effect for TTX-exposed group can be quantitatively related to the ionic correlation time by equation (3). The fractional exponents of a control group showed a strong memory effect which can be interpreted as a longer correlation time of a ionic current. In contrast, the majority of neurons exposed to TTX had a fractional exponent larger than 1.5, which corresponds to $H\leqslant 0.67,$ and subsequently, the rapidly decaying feature of the ionic current was expected. Note that for the fractional exponent *α* = 2 and Hurst exponent at *H* = 0.5, the process loses the memory effect, and becomes Brownian motion. The effect of TTX on blocking the generation of action potentials from the neurons can be interpreted as a reduced memory effect, demonstrated by the label-free, non-invasive, optophysiological method presented here. The fractional exponent interpretation based on the dynamic OCM images can be further considered as spatial correlations that describe neuronal connectivity, and the interactions of ion transport and gating that serve to induce neuronal activity and action potential firing between adjacent cells. The current estimation cannot distinguish the difference between internal or external noise contributions, however, the memory effect altered by localized external perturbations and the propagation of the effect could be used to determine the contributions from each of the internal and external noise sources (Vazquez-Guerrero *et al* 2019).

## 4. Conclusion

In summary, we demonstrated a statistical approach to characterize the non-ergodic neuronal dynamics by a non-invasive, label-free, phase-sensitive optophysiological detection method. Neurons were assumed to be driven by a force induced by ion transport and gating with a memory effect. Within the time window of 0.25 ms, the displacements of neurons were assumed to have small displacement and obey the Lévy process. The estimation of the memory effect was conducted by MLE of experimentally measured PDF and Lévy distribution in equation (2). Cultured neurons have spontaneous electrical activity, and the corresponding membrane dynamics were observed by phase-sensitive OCM. The optical displacements of the neuronal membranes were acquired and the distribution of the displacements had a power-law distribution. The electrophysiological activities of the neurons were measured by a standard micropipette electrode and the corresponding power-law features of the PDFs were shown. Sodium-ion gating across the neuronal membranes was inhibited by a sodium-channel blocker (TTX) and a suppression of the power-law features in the PDFs was observed. The interpretations of the fractional exponent estimated from the Lévy distribution in equation (3) were performed by introducing the Hurst exponent. The Hurst exponent describes the asymptotic features of the correlation time of ionic current and the sojourn time distribution of the close state. Neurons exposed to TTX showed reduced self-similarity, ionic current correlations, and memory effect. From the Hurst exponent, the fractional geometry of the neuronal dynamics and the degree of self-similarity of neuronal connectivity can be further estimated. Non-Markovian interpretation of the neuronal dynamics could broaden the scope of detection feasibility that can be extended to a higher-dimensional analysis beyond the one-dimensional data demonstrated in this report.

## Data Availability

The data that support the findings of this study are available upon request from the authors. (and any supplementary information files).

## Conflict of interest

The authors have no conflicts of interest to disclose.
